# Update Telenotfallmedizin: Status quo und Ausblick

**DOI:** 10.1007/s00101-023-01301-4

**Published:** 2023-06-12

**Authors:** Hanna Schröder, Stefan K. Beckers, Christina Borgs, Rolf Rossaint, Marc Felzen

**Affiliations:** 1 Klinik für Anästhesiologie, Medizinische Fakultät, Uniklinik RWTH Aachen, RWTH Aachen University, Pauwelsstr. 30, 52074 Aachen, Deutschland; 2 Aachener Institut für Rettungsmedizin & zivile Sicherheit, Uniklinik RWTH Aachen, Aachen, Deutschland; 3 Fachbereich Feuerwehr und Rettungsdienst Aachen, Ärztliche Leitung Rettungsdienst, Aachen, Deutschland

**Keywords:** Steigende Einsatzzahlen, Telenotarzt, Ressourcenknappheit im Rettungsdienst, Notfallsanitäter, Notarztindikationskatalog, Increasing mission numbers, Tele-EMS physician, Resource scarcity in EMS, Paramedics, EMS physician indications catalog

## Abstract

**Aktueller Stand der Notfallmedizin in Deutschland:**

In den letzten Jahren kommt es bei steigendem Einsatzaufkommen zu zunehmendem Mangel von nichtärztlichem, aber auch ärztlichem Personal im Rettungsdienst, sodass eine optimierte Nutzung der vorhandenen Ressourcen erforderlich ist. Eine Möglichkeit stellt die Einführung des Telenotarztes (TNA) dar, welcher in Aachen bereits seit 2014 in den Regelrettungsdienst eingebunden ist.

**Einführung von Telenotfallmedizin:**

Neben Pilotprojekten führen Beschlüsse der politischen Entscheidungsträger zur Einführung von Telenotfallmedizin. Der Ausbau schreitet aktuell in unterschiedlichen Bundesländern voran; für Nordrhein-Westfalen und Bayern ist eine flächendeckende Einführung beschlossen. Essenziell für die indikationsgerechte Einbindung von Telenotfallmedizin ist die Anpassung des Notarztindikationskatalogs.

**Status quo von Telenotfallmedizin:**

Der Telenotarzt bietet das Potenzial, (not‑)ärztliche Expertise langfristig und flächendeckend standortunabhängig im Rettungsdienst zu verankern und damit ärztlichen Ressourcenmangel teilweise zu kompensieren. Weiterhin kann er die Leitstelle beratend unterstützen sowie beispielsweise Sekundärtransporte abklären. Ein einheitliches Qualifikationscurriculum für Telenotärzte wurde von den Ärztekammern Nordrhein und Westfalen-Lippe eingeführt.

**Ausblick:**

Neben der Konsultation aus Primär- und Sekundäreinsätzen kann Telenotfallmedizin in weiteren Bereichen eingesetzt werden, beispielsweise zur Supervision von ärztlichem Personal oder Teilen der Rezertifizierung von Rettungsdienstpersonal. Eine Kompensation eines Mangels an Rettungswagen könnte durch den Gemeindenotfallsanitäter erfolgen, welcher ebenfalls an den Telenotarzt angeschlossen werden kann.

## Abstract (en)

**Current status of emergency medicine in Germany:**

Increasing numbers of rescue missions in recent years have led to a growing staff shortage of paramedics as well as physicians in the emergency medical system (EMS) with an urgent need for optimized usage of resources. One option is the implementation of a tele-EMS physician system, which has been established in the EMS of the City of Aachen since 2014.

**Implementation of tele-emergency medicine:**

In addition to pilot projects, political decisions lead to the introduction of tele-emergency medicine. The expansion is currently progressing in various federal states, and a comprehensive introduction has been decided for North Rhine-Westphalia and Bavaria. The adaptation of the EMS physician catalog of indications is essential for the integration of a tele-EMS physician.

**Status quo of tele-emergency medicine:**

The tele-EMS physician offers the possibility of a long-term and comprehensive EMS physician expertise in the EMS regardless of location and, therefore, to partially compensate for a lack of EMS physicians. Tele-EMS physicians can also support the dispatch center in an advisory capacity and, for example, clarify secondary transport. A uniform qualification curriculum for tele-EMS physicians was introduced by the North Rhine and Westphalia-Lippe Medical Associations.

**Outlook:**

In addition to consultations from emergency missions, tele-emergency medicine can also be used for innovative educational applications, for example, in the supervision of young physicians or recertification of EMS staff. A lack of ambulances could be compensated for by a community emergency paramedic, who could also be connected to the tele-EMS physician.

## Aktueller Stand der Notfallmedizin

Aktuellen Entwicklungen folgend nimmt der Personalmangel sowohl des ärztlichen als auch des nichtärztlichen Personals im Rettungsdienst zu. Zusätzlich steigen die Qualifikationsanforderungen aufgrund des Notfallsanitätergesetzes [18], der Zusatzweiterbildung Notfallmedizin [21] und einer ärztlichen Fortbildungsverpflichtung [29]. Rettungsmittel können zunehmend nicht mehr besetzt werden. Während die Schwelle zur Alarmierung des Rettungsdienstes aufgrund eines höheren Anspruchsdenkens der Bevölkerung einerseits sinkt [4, 27, 28], nimmt andererseits das Absicherungsbedürfnis des Leitstellenpersonals und auch Rettungsdienstes aufgrund einer zunehmenden Klagebereitschaft zu [27]. Daraus resultierend wird eine stetig ansteigende Inanspruchnahme des Rettungsdienstes nahezu bundesweit verzeichnet. Das Zusammenspiel dieser Faktoren macht eine optimierte Nutzung der vorhandenen Ressourcen zwingend erforderlich, um das notwendige Qualitätsniveau bei erhöhtem Einsatzaufkommen halten oder gar steigern zu können.

An dieser Stelle kommt der Telenotfallmedizin als ergänzende Ressource im Rettungsdienst eine besondere Bedeutung zu. Sie kann eine notwendige, jedoch personell nicht kompensierbare Aufstockung arztbesetzter Rettungsmittel durch Übernahme bestimmter Notarzteinsätze abmildern. Dies führt zu einem indikationsgerechteren Einsatz der arztbesetzten Rettungsmittel.

## Einführung von Telenotfallmedizin

Die Telenotfallmedizin kann ökonomisch sinnvoll ergänzend zum bisher bestehenden Rettungsdienstsystem eingeführt werden, wenn die Notfallversorgung der Bevölkerung an ihre Leistungsgrenze stößt, sodass mit Qualitätseinbußen zu rechnen ist.

Bei der Implementierung von Telenotfallmedizinsystemen stehen unterschiedliche technische Ausbaustufen und Anbieter zur Verfügung, vom einfachen Telefonat („call back“) bis hin zu integrierten Systemkomponenten (Tab. 1) mit Übertragung sämtlicher an der Einsatzstelle erhobener Befunde durch Echtzeitvitaldaten, Bild, Ton und Video [1, 19]. Allerdings ist der Begriff „Telenotarzt“ (Tab. 1) angelehnt an die S1-Leitlinie der Deutschen Gesellschaft für Anästhesiologie und Intensivmedizin (Telemedizin in der prähospitalen Notfallmedizin) im Rahmen des Implementierungsprozesses in Nordrhein-Westfalen (NRW) definiert als *ein im Rettungsdienst eingesetzter, speziell geschulter und erfahrener Notarzt, der mittels Sprach- und Sichtkontakt, Echtzeit-Vitaldaten und Videoübertragung gemeinsam mit dem Rettungsteam vor Ort Notfallpatienten im Regelrettungsdienst versorgt. *Darüber hinaus ist ein Telenotarzt nicht einfach als Arzt am Telefon zu verstehen, sondern es Bedarf der Erfüllung bestimmter Voraussetzungen. So ist z. B. zu unterscheiden, ob ein zusätzlicher Arzt rund um die Uhr an 7 Tagen der Woche an einem speziell dafür abgestimmten Arbeitsplatz verfügbar ist, oder ob ein im Regeldienst befindlicher Notarzt in der einsatzfreien Zeit oder aus dem Auto Anrufe und Nachfragen von Rettungsdienstbesatzungen bedient.

**Table 1:** 

Telenotarzt (TNA)	Ein im rettungsdienstlich qualifizierter, erfahrener und speziell geschulter Notarzt, der mithilfe von Telekommunikation, Echtzeitvitaldatenübertragung, Sprach- sowie ggf. Sichtkontakt das Rettungsfachpersonal vor Ort unterstützt und so Patienten im Regelrettungsdienst versorgt
Telenotarztsystem (TNA-System)	Leitlinienorientiertes Konzept eines „holistischen“ Systems (Anamnese, Dokumentation, Einsatzbearbeitung in einem System) unter Berücksichtigung der Anforderungen des Datenschutzes, der Dokumentationsqualität, technischer Standards, der Rechtssicherheit sowie definierter Qualitätsmerkmale durch den ÄLRD
Telenotarztzentrale (TNAZ)	Standorteinheit des TNA mit Zugriff auf das TNA-System zur Wahrnehmung der Aufgaben des TNA-Diensts, die für den Standort durch den oder die zuständigen ÄLRD festgelegt wurden; Anbindung an Einsatzleitsystem der Leitstelle
Technische Systemkomponenten eines TNA-Systems am Beispiel des Systems Aachen	Stationäre und mobile Fahrzeugtechnik (zentrales Kommunikationssteuerelement, Hardware zur bidirektionalen Kommunikation), kompatible Medizintechnik zur Übertragung von Echtzeitvitaldaten sowie 12-Kanal-EKG, TNA-Zentrale mit Logistik und Hardware, Software des TNA-Systems inkl. nötiger Schnittstellen, verteilte Serverumgebung

*ÄLRD* Ärztlicher Leiter Rettungsdienst, *EKG* Elektrokardiogramm

Bei der Einführung ist sowohl die Disposition durch die Leitstelle (z. B. Modell Greifswald) als auch die direkte Konsultation durch Rettungsdienstpersonal ohne vorherige Alarmierung möglich (z. B. Modell Aachen). Konsultiert Rettungsdienstpersonal, so kann eine Konsultation ohne Einbindung eines Notarztes, während seiner Anfahrt oder aber mit seiner Nachalarmierung erfolgen. Die Vor- und Nachteile beider Methoden sind in Tab. 2 dargestellt.

**Table 2:** 

Einbindung des TNA	Disposition durch Leitstelle	Anforderung durch Rettungsteams lt. SOP
Vorteile	Garantierte ärztliche Beurteilung der Patient*innen	Konsultation nur bei durch Rettungsteam identifizierter Indikation (Filterung abfragebedingter Unschärfe)
Nachteile	Alarmierungen bei vor Ort nicht bestehender Arztindikation	Ausbleibende ärztliche Beurteilung bei fehlender Konsultation trotz Indikation

Für beide dargestellten Einbindungsoptionen gilt, dass eine Reduzierung absoluter Notarztindikationen erfolgen muss

Um eine effiziente und ressourcengerechte Implementierung von Telemedizinsystemen zu realisieren, ist die Bildung von Trägergemeinschaften ähnlich wie bei Luftrettungsmitteln erforderlich. Zur Schaffung einer uneingeschränkten Verfügbarkeit von Telenotfallmedizin bei Duplizität (d. h. zeitgleiches Auftreten von Einsätzen in einem Versorgungsgebiet) sollten, analog zum Regelrettungsdienst, Redundanzen in Form mehrerer Arbeitsplätze in einer Zentrale, einer Vernetzung mehrerer Telenotarztzentralen sowie einer Standardisierung der Qualifikation des Personals [16, 38] geschaffen werden.

Gemäß einer durch die Universität Maastricht (NL) durchgeführten Potenzialanalyse ist ein Telenotarztarbeitsplatz in der Lage, ca. 1–1,5 Mio. Einwohner in Form einer Trägergemeinschaft zu versorgen [31].

## Status quo von Telenotfallmedizin

### Telenotarztzentrale der Stadt Aachen, Nordrhein-Westfalen

**Figure 1:**
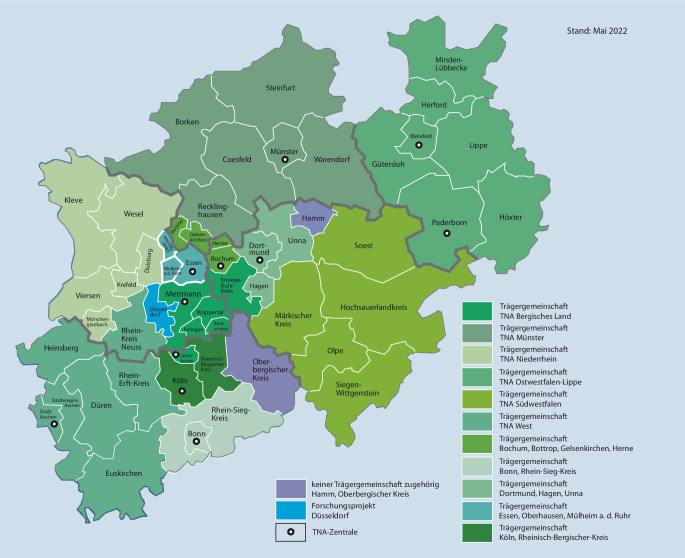

**Figure 2:**
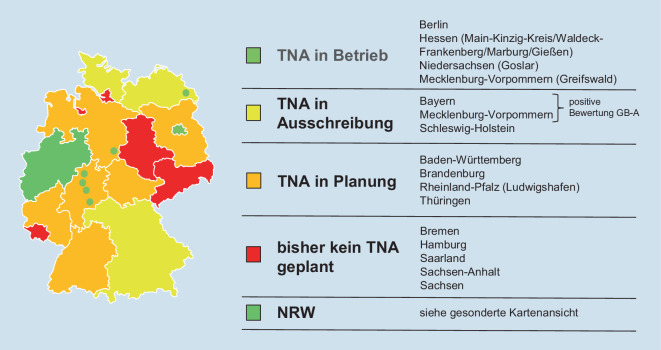

Nach anfänglicher Skepsis vieler Experten der prähospitalen Notfallmedizin werden telenotfallmedizinische Lösungen nach den positiven Erfahrungen des Telenotarztsystems der Stadt Aachen seit dem Jahre 2014 aktuell an immer mehr Standorden in der Bundesrepublik etabliert. Hintergrund ist ein an einigen Standorten bereits manifester Notarztmangel mit daraus resultierenden verlängerten Eintreffzeiten [26]. Dieser hat in Nordrhein-Westfalen zu einem Beschluss der politischen Entscheidungsträger im Einvernehmen mit den Kostenträgern, den kommunalen Spitzenverbänden und den Ärztekammern zur flächendeckenden Einführung von Telenotfallmedizin beigetragen (Abb. 1, [19]). Andernorts erfolgt die Einführung von Telenotfallmedizin bzw. derartigen Projekten jedoch auch ohne derartige Beschlüsse (Abb. 2).

### Telemedizinischer Ausbau in Deutschland

Bisher ist nur in wenigen Bundesländern kein telemedizinischer Ausbau geplant (Abb. 2). Für Bayern und Mecklenburg-Vorpommern liegt eine positive Bewertung des gemeinsamen Bundesausschusses vor. Der Bayerische Ministerrat war 2019 der erste, der eine landesweite Einführung von Telemedizin beschlossen hat. Der künftige Betreiber der ersten von 3 geplanten Standorten ist identifiziert, die künftige Telenotarztzentrale im Rettungsdienstbereich Straubing-Bogen ist baulich fertiggestellt, und auch der Systemanbieter wurde mittels Ausschreibung ausgewählt. Ein Betriebsbeginn ist noch für 2023 zu erwarten. Telenotfallmedizinsysteme in zahlreichen weiteren Bundesländern sind in Planung oder bereits eingeführt bzw. in Form von Telemedizinprojekten etabliert.

### Qualifikation der Telenotärzt:innen

Neben technischen Schnittstellen zwischen den Systemanbietern ist zur Gleichbehandlung aller Patient:innen eine standardisierte Qualifikation aller Telenotärzt:innen erforderlich, welche deutlich über die einer Notärztin/eines Notarztes hinausgeht und spezielle Fertigkeiten und Expertise im Rettungsdienst erfordert. Ein erstes „Qualifikationscurriculum Telenotarzt“ wurde im März 2021 gemeinsam durch die Ärztekammern Westfalen-Lippe und Nordrhein auf Facharztniveau eingeführt, und ein zugehöriges Zertifikat wird bislang in Deutschland nur durch diese beiden Ärztekammern vergeben [3]. Diese Qualifikation garantiert ein vergleichbares Ausbildungsniveau der Telenotärzt:innen, denn sie müssen in der Lage sein, Notfallpatient:innen aus der Ferne anhand weniger zur Verfügung stehender Informationen richtig einzuschätzen sowie Verantwortung zu übernehmen, ohne Patient:innen gegenüberzustehen [16]. Vertrauen in häufig unbekannte Teams vor Ort ist essenziell, da ein manuelles Eingreifen nicht möglich ist. Darüber hinaus sind Delegationsfähigkeit und Führungskompetenz unabdingbare Voraussetzungen, um als Telenotarzt erfolgreich zu sein. Parallelkonsultationen aus unterschiedlichen Rettungsdienstbereichen (in der Aachener Telenotarztzentrale im Mittel bei jedem 4. Einsatz) setzen Stressresistenz sowie Multitasking-Fähigkeiten voraus. Eine strukturierte Übergabe zu Konsultationsbeginn, welche sofort mitdokumentiert wird, erfordert zudem eine hohe Konzentrationsfähigkeit, insbesondere auch bei nächtlichen Einsätzen ohne Vorlaufzeit.

### Möglichkeiten mithilfe von Telenotfallmedizin

Ziel von Telenotfallmedizin ist es, per Knopfdruck (tele)notärztliche Expertise und rechtssichere Entscheidungskompetenz über die Entfernung hinweg (mit uneingeschränktem Zugriff auf aktuelle Leitlinien) an eine oder sogar parallel mehrere Einsatzstellen zu bringen. Dadurch soll die Qualität der Patientenversorgung gehalten oder gar verbessert werden (Tab. 3).

**Table 3:** 

Qualitätsverbesserung	Erläuterung
Indikationsgerechterer Einsatz des Notarztes	Rechtssichere Übernahme sämtlicher Aufgaben des Notarztes, welche die manuellen Fertigkeiten des Arztes vor Ort nicht erfordern
Umfassende Dokumentation	Vollständige und ausführliche, digitale Dokumentation bedingt durch Abstand zur Einsatzstelle sowie keinerlei manueller Aufgaben
Unterstützung der Notfallsanitäter:innen	Jederzeitige Konsultation eines Arztes auf Knopfdruck
Sekundärtransporte	Unmittelbare Abklärung via Arzt-Arzt-Gespräch mit anschließender Ressourcenauswahl, Möglichkeit der telemedizinischen Begleitung von Sekundärtransporten
Leitlinienadhärenz	Softwarebasiertes Anzeigen aller aktuellen Leitlinien, inklusive Checkliste, auf Knopfdruck, sodass auch seltenere Krankheitsbilder leitliniengetreu behandelt werden können
Telenotarztkonsultation komplikationsarm	Die Komplikationsrate des Telenotarztes ist nicht höher als die des Notarztes
Telenotarztkonsultation rechtssicher, keine Fernbehandlung	Gutachten belegen die Rechtssicherheit der Telekonsultation und bestätigen, dass es sich hierbei nicht um eine Fernbehandlung handelt, da dem Arzt alle benötigten Informationen und Vitalwerte zur Verfügung stehen
Trägergemeinschaften möglich	Ein Telenotarztarbeitsplatz kann eine hohe Anzahl an Rettungswagen versorgen, Trägergemeinschaften sind aufgrund der Ortsunabhängigkeit gut realisierbar
Einheitliche Qualifikation	Ein Qualifikationscurriculum der Ärztekammern Nordrhein und Westfalen-Lippe ermöglicht eine einheitliche Qualifikation aller Telenotärzte auf Facharztniveau
Paralleleinsätze	Mehrere Konsultationen parallel möglich, während ein NEF immer nur einen Einsatz übernehmen kann
Vernetzung von Telenotarztzentralen	Schaffung von Redundanzen bei Auslastung einer Telenotarztzentrale
Supervision neuer Notärzt:innen	Telenotarzt hört Kommunikation und sieht sämtliche Vitalparameter, neue Kolleg:innen erhalten nach dem Einsatz Feedback, jedoch ist auch während des Einsatzes jederzeit eine Kommunikation bzw. ein Eingreifen durch den Telenotarzt möglich
Rezertifizierung von Notfallsanitäter:innen	Rezertifizierung im Realeinsatz ohne Übungskünstlichkeit, Notfallsanitäter:in arbeitet den Einsatz selbstständig ab, Telenotärzt:in greift nur bei Patientengefährdung ein, im Anschluss strukturierte Bewertung durch den Telenotärzt:in

Das Telenotarztsystem der Stadt zeigt eine zuverlässige technische Performance der einzelnen Komponenten bei einer Ausfallquote einzelner Komponenten von 2 % (Audioverbindung) bis maximal 7 % (12-Kanal-EKG). Komplettausfälle des Systems waren mit 0,6 % (*n* = 3) im Untersuchungszeitraum sehr selten [15]. Seine Vorteile sind zusammengefasst in Tab. 3 dargestellt. Im Rahmen einer in Aachen durchgeführten Dreijahresanalyse des Telenotarztsystems konnte gezeigt werden, dass der Telenotarzt dem konventionellen Notarzt hinsichtlich der Komplikationsrate nicht unterlegen ist [13]. Ähnliche Ergebnisse werden im Rahmen der in Aachen durchgeführten randomisierten kontrollierten TEMS-Studie [36] erwartet. Weitere Untersuchungen belegen den sicheren und komplikationsarmen Einsatz von Telenotfallmedizin (Tab. 4).

**Table 4:** 

Kernaussagen	Literaturverweis
*Telenotarzt ist konventionellem Notarzt* bei nicht akut lebensbedrohlichen Notfällen *nicht unterlegen; Reduktion des arztfreien Intervalls* (Forschungsschwerpunkte: hypertensiver Notfall, Monotrauma, ACS, Stroke)	[5–7, 10, 11, 13]
Telekonsultation bei *lebensbedrohlichen Umständen* ist sinnvoll möglich und führt zur Stabilisierung kritischer Vitalwerte; führende Krankheitsbilder mit Notarztnachforderung: STEMI, kardiales Lungenödem, Herzrhythmusstörungen mit Instabilitätskriterien	[32]
Frühere und gleichermaßen erfolgreiche Analgesie durch den Telenotarzt (Standortvergleich: Morphin auch im „call back“ suffizient möglich)	[20, 24]
Sehr gute *technische Performance* des Systems	[8, 15]
Geringe Nutzung der Videokamera (jedoch gehäuft bei respiratorischen Störungen, Reposition)	[13, 30]
*Pandemie*: Reduktion nichtindizierter Krankenhaustransporte bei stabilen COVID-Patienten durch TNA-Konsultation	[14]
Die optimierte Nutzung der vorhandenen Ressourcen führt zu einer *höheren Verfügbarkeit des konventionellen Notarztes* für wirklich akut lebensbedrohliche Notfälle wie Bewusstlosigkeit, akute Atemnot, Herz-Kreislauf-Stillstand oder Polytraumatisierung, welche die manuellen Fertigkeiten des Notarztes erfordern	[13]
Die zusätzliche Ressource TNA führt zu einer *deutlichen Reduzierung* ärztlich begleiteter *Sekundärtransporte*, sowohl durch telemedizinische Begleitung als auch durch strukturierte vorherige Abklärung	[34]

*ACS* Akutes Koronarsyndrom, *STEMI* ST-Elevation Myocardial Infarction, *TNA* Telenotarzt

### Anpassung des Notarztindikationskatalogs

Entscheidend für die Einbindung der Telenotfallmedizin in einem Rettungsdienstbereich ist die Anpassung des Notarztindikationskatalogs. Dazu stehen sowohl die aktuell in der Aktualisierung befindliche S1-Leitlinie „Telemedizin in der prähospitalen Notfallmedizin“ [39] als auch zukünftig ein Indikationskatalog Telenotarzt NRW zur Verfügung. Der lokal verwendete Notarztindikationskatalog (NAIK) wird in zahlreichen Bundesländern von der Ärztlichen Leitung Rettungsdienst (ÄLRD) festgelegt und verantwortet. Orientierende Grundlage ist der Notarztindikationskatalog der Bundesärztekammer von 2013 [2], der jedoch aktuelle Weiterentwicklungen im Rettungsdienst wie u. a. das Notfallsanitätergesetz (NotSanG) bislang nicht berücksichtigt und sich deswegen zurzeit noch in Überarbeitung befindet [40]. Die Bundesländer setzen diesen jedoch unterschiedlich um. So gilt beispielsweise in Hessen nach Beschluss der dort tätigen ÄLRD ein angepasster Notarztindikationskatalog mit Berücksichtigung zustandsbezogener Indikationen (mit vitaler Bedrohung) sowie situationsbezogener Indikationen aufgrund von anzunehmender Lebensgefahr [22].

Aufgrund von Interpretationsspielraum sowie unterschiedlichem Sicherheitsbedürfnis der ÄLRD führt dies in der BRD unabhängig von der Verfügbarkeit von Telenotfallmedizin zu heterogenen Alarm- und Ausrückeordnungen (AAO) und somit zu einer großen Spanne der Notarztquoten (definiert als Anteil der Notarzteinsätze an den Gesamtrettungsdiensteinsätzen) von 16–57 % [9, 31]. Folglich kann bereits die alleinige Überarbeitung des Notarztindikationskatalogs zu einer optimierten Nutzung der vorhandenen Ressourcen führen. Einbindungsmöglichkeiten von Telenotfallmedizin in die AAO sowie damit verbundene Vor- und Nachteile sind in Tab. 2 dargestellt.

**Figure 3:**
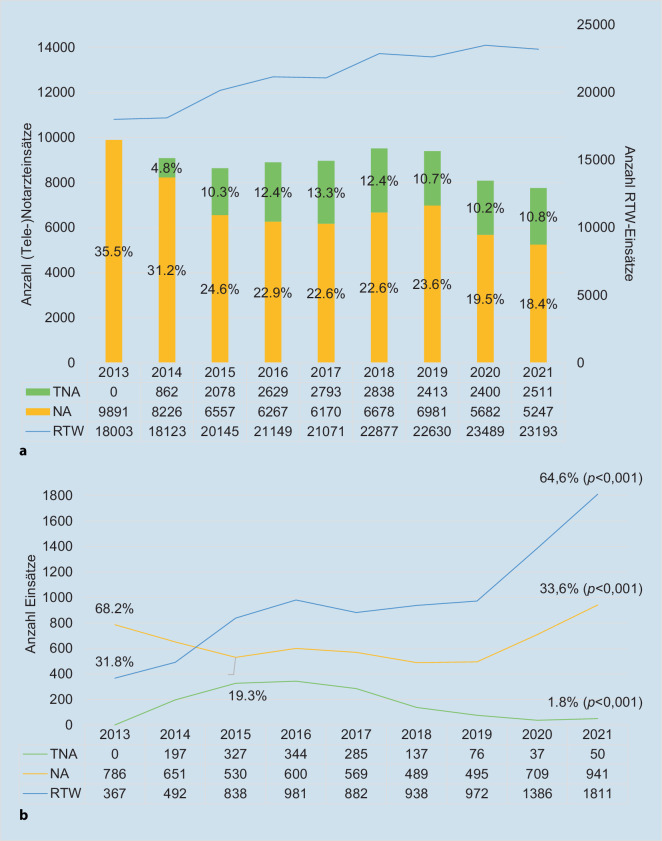

In Aachen hat die mit der Einführung der Telenotfallmedizin verbundene Anpassung des Notarztindikationskatalogs zwischen 2014 und heute zu einer Reduktion der Notarztquote um 50 %, d. h. absolut von 36 auf 18 % geführt (Abb. 3).

So wurde beispielweise in Aachen das Meldebild des Akuten Koronarsyndroms (AKS) aufgeteilt in ein Meldebild mit Notarzt (AKS kaltschweißig, blass) und ein Meldebild ohne Notarzt (AKS nicht kaltschweißig). Entscheidet sich der Disponent für ein Meldebild ohne *akut* lebensbedrohliches Krankheitsbild, wird kein bodengebundener Notarzt, sondern alleinig ein Rettungswagen (RTW) mit Möglichkeit zur Telekonsultation entsandt, welcher gemäß Verfahrensanweisungen oder eigenem Ermessen den Telenotarzt konsultieren kann.

Rund ein Viertel der früheren Notarzteinsätze werden durch den Telenotarzt übernommen und können in über 92 % ohne Beteiligung eines Präsenznotarztes vollständig abgearbeitet werden. Außerdem wird jeder Fünfte der ehemaligen Notarzteinsätze nach Anpassung der Notarztindikationen heute durch die Rettungsteams alleine abgearbeitet. Eigenverantwortliche Maßnahmen der Notfallsanitäter gemäß §4 Abs. 2, Nr. 2C NotSanG sind in der Stadt Aachen seit April 2022 umgesetzt und werden in Zukunft zu einer weiteren Steigerung der alleinigen Abarbeitung von Einsätzen durch Rettungsteams führen.

Anfängliche Bedenken bezüglich der „Wegrationalisierung“ des bodengebundenen Notarztes durch die Telenotfallmedizin werden bei genauer Betrachtung des Systems zunehmend entkräftet. Unstrittig ist, dass der konventionelle Notarzt für spezielle Notfallbilder auch zukünftig erforderlich ist. Es kann jedoch weder der Anspruch eines Rettungsdienstträgers noch der Notärzt:innen selbst sein, eine derart wertvolle sowie immer schwieriger verfügbare Ressource nicht indikationsgerecht einzusetzen, ganz besonders in Zeiten zunehmender Kompetenz von Notfallsanitäter:innen.

### Einsatzspektrum der Telenotarztzentrale Aachen

Zwischen dem 01.04.2014 und dem 31.03.2022 konnten durch die Telenotarztzentrale Aachen insgesamt 28.705 Primäreinsätze und 1871 Sekundäreinsätze in allen aufgeschalteten Bereichen (Stadt Aachen, Städteregion Aachen, Kreis Borken, Kreis Düren, Kreis Euskirchen, Kreis Heinsberg, Main-Kinzig-Kreis, Kreis Waldeck-Frankenberg, Halligen Hooge und Langeness) telenotfallmedizinisch unterstützt werden.

Mit der Konsultation durch den Rettungswagen wird der Telenotarzt Teil des Behandlungsteams für den vorliegenden Einsatz. Sein Aufgabenspektrum umfasst die Einordnung der verfügbaren Anamneseinformationen und Befunde (inkl. EKG-Diagnostik), das Stellen einer Verdachtsdiagnose, die Initiierung von Therapiemaßnahmen wie beispielweise Medikamentengaben, Entscheidungsfindung und Auswahl des richtigen Transportziels sowie Voranmeldung im aufnehmenden Krankenhaus.

Das Einsatzspektrum geht weit über die in der Leitlinie aufgeführten Indikationen hinaus. Der häufigste Konsultationsgrund ist die Analgesie sowohl bei traumatisch als auch nicht traumatisch bedingten Schmerzen, welche jedoch gerade einmal knapp ein Drittel der Gesamtkonsultationen ausmachen. Konsultationen erfolgen bei in Standardarbeitsanweisungen (SAA) niedergeschriebenen Tracer-Diagnosen teilweise bedingt durch Vorgaben, aber auch bei unklaren, nicht einer Tracer-Diagnose zuzuordnenden Symptomen. Selbst bei lebensbedrohlichen Notfällen kann die Telenotarztkonsultation den Zustand des Patienten bis zum Eintreffen des Notarztes verbessern [32]. Während der Coronapandemie konnten nichtindizierte Krankenhaustransporte bei stabilen COVID-Patient:innen durch eine Telenotarztkonsultation ärztlich abgesichert werden [14].

Durch die Anbindung der Halligen-Marschinseln Hooge und Langeness in Schleswig-Holstein gehören auch hausärztliche Fragestellungen zum Einsatzspektrum am Arbeitsplatz. Diese reichen von Verordnung einer Antibiose oder Hautsalbe bis hin zum Erfordernis der Alarmierung eines Rettungshubschraubers oder Seenotkreuzers zwecks dringlichem Abtransport. Dadurch wird deutlich, dass der Übergang zwischen Telenotfallmedizin und Telemedizin fließend sein kann. Zum anderen beweist diese Speziallösung, dass es sich bei Telemedizin um ein breites Anwendungsfeld handelt.

Über die Laufzeit des Telenotarztsystems haben sich neben den Primäreinsätzen weitere Einsatzbereiche und Aufgabenfelder wie Übernahme der Informationseinholung bei der Giftnotrufzentrale, überbrückende Konsultation bis zum Eintreffen des (nach-)alarmierten Notarztes, Zweitmeinung für einen Notarzt vor Ort, Übernahme der Transportbegleitung für einen Notarzt oder einen Sekundärtransport sowie Beratung der Leitstelle etabliert.

### Der für die Leitstelle verfügbare Arzt

Neben dem breiten Einsatzspektrum in der primären Notfallrettung kann ein Telenotarzt weitere Aufgaben, welche zur Ressourcenschonung beitragen können, übernehmen. Beispielsweise werden Sekundärtransporte in vielen Rettungsdienstbereichen mangels verfügbarer ärztlicher Abklärungsmöglichkeit wie vom Krankenhaus angefordert – und damit häufig zeitnah und mit Notarztbegleitung – disponiert. Die ständige Verfügbarkeit eines Telenotarztes bietet die Möglichkeit einer unmittelbaren Abklärung in einem strukturierten Arzt-Arzt-Gespräch, auch wenn dies per se keine Telemedizin erfordert. Dadurch lassen sich unnötige Arztbegleitungen reduzieren und die angemessene Personal- und Materialressource auswählen [34]. Voraussetzung ist ein ausreichend geschultes Personal. So haben Schulungen des nichtärztlichen Rettungsdienstpersonals im Umgang mit tracheotomierten Patienten in Aachen zu einer Reduktion der arztbegleiteten Verlegungen von 68,2 % (*n* = 786; 2013) auf 33,6 % (*n* = 941; 2021) geführt (Abb. 3). Verlegungen mit Primärnotarzt und Notarzteinsatzfahrzeug (NEF) sind von 20,1 % (*n* = 292; 2013) auf 3,5 % (*n* = 97; 2021) gesunken.

Nachdem anfänglich aufgrund eines erhöhten Sicherheitsbedürfnisses Sekundärtransporte anstatt mit Notarzt mit Telenotarztkonsultation durchgeführt wurden, werden Sekundärtransporte nach strukturierter ärztlicher Abklärung zunehmend alleine durch RTW-Teams übernommen. Unkenntnis hinsichtlich Personal, Ausstattung und Möglichkeiten von v. a. Krankentransportwagen (KTW) besteht oftmals selbst bei im Rettungsdienst erfahrenen Ärzten, dabei sind KTW als Ressource nicht zu vernachlässigen.

Gemäß Rettungsdienstgesetz Nordrhein-Westfalen hat der Träger des Rettungsdienstes einen Sicherstellungsauftrag vorrangig für die Notfallrettung. Demgegenüber steht die vielerorts noch übliche Alarmierung von v. a. ärztlichen Ressourcen der Primärrettung für disponible Sekundärtransporte. Dies führt oft zu einer unnötigen Unterdeckung von Rettungsdienstbereichen, welche mit einer potenziellen Patientengefährdung einhergeht. Aus diesem Grund besteht hier Nachsteuerungsbedarf. Es ist nicht sinnvoll, eine Arztindikation nur anhand einer Diagnose festzulegen, vielmehr sollte der aktuelle Zustand des Patienten zusätzlich Berücksichtigung finden. Auch hilft bei dieser Entscheidung der Krankenhausarzt nicht unbedingt weiter, wenn dieser die materiellen und personellen Ressourcen des Rettungsdienstes nicht überblickt. Da es für einen Leitstellendisponenten oftmals schwierig ist, gegen die Entscheidung des anfordernden Krankenhausarztes zu argumentieren, sollte sinnvollerweise ein direktes und strukturiertes Arzt-Arzt-Gespräch erfolgen und auf der Grundlage dessen sollten die geeigneten Rettungsmittel durch einen im Rettungsdienst erfahrenen Arzt ausgewählt werden. Dieser Arzt kann praktikabelerweise, muss jedoch nicht, der Telenotarzt sein.

### Qualitätsmanagement im Rahmen der Telenotarztimplementierung

Qualitätsmanagement [33] ist im Rettungsdienst unerlässlich. Hintergrund ist die Erfüllung von Qualitätsindikatoren, wie z. B. im Eckpunktepapier Notfallversorgung [17] beschrieben. Enorm hilfreich bei der Datenakquise und strukturierten Analyse ist eine digitale Dokumentation, welche jedoch im Rettungsdienst bisher nur zu ca. 35 % eingeführt ist [25]. Die standardisierte digitale Dokumentation in Telenotarztsystemen (Tab. 1) erleichtert die Datenzusammenführung von Rettungsmittel und Telenotarztdienst. Eine gleichwertige Datenbasis ist mit papierbasierter Dokumentation aufgrund des erheblichen Aufwands der Digitalisierung praktisch nicht erreichbar. Darüber hinaus hat die Einbindung des Telenotarztes (im Sinne der Definition aus Tab. 1) in einigen Rettungsdienstbereichen erst dazu geführt, dass vorher nichtvorhandene Verfahrensanweisungen etabliert wurden.

Mit Einführung eines Telenotfallmedizinsystems sollte ein effektives Qualitätsmanagementsystem [33] etabliert werden.

### Der Telenotarzt und das Notfallsanitätergesetz

Das Telenotarztsystem gerät immer wieder in die Kritik von Interessensverbänden des Rettungsfachpersonals, mit der Argumentation, den Notfallsanitäter:innen die Möglichkeit eines eigenverantwortlichen Handels zu nehmen. Zweifelsohne steht eine jederzeit verfügbare Konsultation eines Arztes aus juristischer Sicht über dem eigenständigen Handeln von nichtärztlichem Personal; nachvollziehbar und konsequenterweise ist selbst ärztliches Personal bei Unsicherheit zur Konsultation angehalten [12]. Dennoch ist es sinnvoll und erforderlich, auch in Rettungsdienstbereichen mit Telekonsultationsmöglichkeit Maßnahmen gemäß §4 Abs. 2 Nr. 2c NotSanG für Notfallsanitäter:innen zu definieren. Diese Maßnahmen bedürfen der regelmäßigen praktischen Anwendung, um im Falle von technischen Problemen, Auslastung des Telenotarztes oder anderer Notwendigkeit beherrscht zu werden, damit die Patient:innenversorgung auch in diesen Fällen nicht leidet. Sollte es bei alleiniger Durchführung zu unvorhersehbaren Ereignissen, Problemen, unerwünschten Wirkungen oder Unsicherheit seitens der Notfallsanitäter:innen kommen, so kann der Telenotarzt jederzeit ohne Zeitverzögerung zur Hilfestellung hinzugezogen werden. Hierfür stehen redundante Kommunikationsmöglichkeiten zur Verfügung. Auch mögliche Bedenken junger Notfallsanitäter:innen beim erstmaligen, alleinigen Durchführen von sog. 2c-Maßnahmen lassen sich durch eine kurze telenotärztliche Konsultation schnell abfangen.

Darüber hinaus können Standardarbeitsanweisungen (SAA) nicht alle denkbaren Symptome und Konstellationen abbilden, stellen jedoch die Voraussetzung und Grundlage für die eigenständige Durchführung von Maßnahmen durch Notfallsanitäter:innen dar. Neben vorgenannter Delegation ist mithilfe eines Telenotarztsystems zusätzlich eine Individualdelegation von Maßnahmen möglich. Die Realität zeigt eine Zunahme der Einsätze mit Arztkontakt seit Einführung des Telenotarztes. Das heißt, dass der Telenotarzt niedrigschwellig bei Fragen konsultiert wird, für welche kein Notarzteinsatzfahrzeug nachgefordert werden würde. Dies führt einerseits zu einem Lerneffekt der Rettungsteams, andererseits zu einer Qualitätssteigerung der Patient:innenversorgung.

## Ausblick

Die deutschlandweite Ausbreitung von Telenotfallmedizin hat bereits begonnen (Abb. 2). Bereits zwei durch den Gemeinsamen Bundesausschuss (G-BA) positiv bewertete Innovationsfondsprojekte in Bayern („Telenotarzt Bayern“) [9] und Mecklenburg-Vorpommern („Land|Rettung“) [41] haben zum Beschluss der landesweiten Einführung von Telenotfallmedizin geführt. Alle weiteren in den Bundesländern für den Rettungsdienst zuständigen Ministerien wurden durch den G‑BA aufgefordert, eine landesweite Einführung zu prüfen.

### Supervision von ärztlichem und nichtärztlichem Personal

Die Telenotfallmedizin bietet ein enormes Potenzial auch über die Konsultation im Primär- oder im Sekundäreinsatz hinaus. So ist beispielsweise die Supervision von Notärzt:innen zu Beginn ihrer Tätigkeit (wie sie aktuell im Rahmen des Projekts COMPAS [42] durchgeführt wird) genauso wie in regelmäßigen Abständen auch nach langjähriger Tätigkeit aus Sicht der Qualitätssicherung denkbar. Hiermit ist eindeutig nicht die Substitution einer dringend erforderlichen Einarbeitung nach Erlangung der Zusatzbezeichnung Notfallmedizin der Ärztekammer gemeint. Die Einarbeitung erfolgt in Aachen nach einem standardisierten Konzept über 5 Werktage durch einen erfahrenen leitenden Notarzt. Die Supervision durch den Telenotarzt erfolgt zusätzlich in den ersten Diensten. Auch die Übernahme von Teilen der Rezertifizierung von Notfallsanitäter:innen durch den Telenotarzt während Realeinsätzen ohne Übungskünstlichkeit ist denkbar.

### Vernetzung von Tele(hausarzt)medizin und Telenotfallmedizin

Weiterhin könnte eine rund um die Uhr, an 7 Tagen in der Woche mögliche Telekonsultation durch z. B. entsprechend ausgestattete Pflegeheime Rettungsdiensteinsätze bzw. Krankenhauseinweisungen verhindern, da es dadurch einen zeitnah verfügbaren Ansprechpartner fernab der Rettungsleitstelle gibt. Dieser Fragestellung widmet sich das aktuell laufende G‑BA-Projekt Optimal@NRW [43].

### Gemeindenotfallsanitäter

Während in Rettungsdienstbereichen mit Telenotarztverfügbarkeit die Ressource Notarzt für Einsätze, bei denen die manuellen Fertigkeiten des Arztes vor Ort erforderlich sind, zurückgehalten werden kann, sind bei steigenden Einsatzzahlen des Rettungsdienstes auch Maßnahmen hinsichtlich eines indikationsgerechteren Einsatzes von Rettungswagen und Krankenwagen erforderlich. Diese sollten alle an der Notfallversorgung beteiligten Institutionen wie z. B. Hausärzte und den ärztlichen Bereitschaftsdienst berücksichtigen. Der Gemeindenotfallsanitäter [35] mit Konsultationsmöglichkeit des Telenotarztes soll hierbei zukünftig eine Schlüsselposition einnehmen. Er soll anstelle bisher eines Rettungswagens immer dann durch die Leitstelle alarmiert werden, wenn am Telefon unklar bleibt, ob vor Ort eine Notfallsituation vorliegt. In Abhängigkeit vom Patientenzustand vor Ort kann er dann bei Bedarf mit Unterstützung des Telenotarztes entscheiden, ob ein Transport indiziert ist und, wenn ja, mit welchem Rettungs- oder Transportmittel dieser stattfinden soll.

### Telenotfallmedizin in Projekten

Die zukünftigen Möglichkeiten der Anwendung von Telenotfallmedizin im Kontext von 5G-Technologien entlang der Rettungskette (wie z. B. die Liveübertragung von Ultraschallbildern) werden derzeit in den Förderprojekten „5G-Telerettung“ (gefördert durch das Bundesministerium für Digitales und Verkehr) sowie „5URVIVE“ (*5*G‑basierte *u*mfassende Strategie zu*r V*erbesserung des Überlebens *i*n der Notfall*ve*rsorgung, gefördert durch das Land NRW) erforscht und entwickelt [37, 44]. Auch die Einsatzmöglichkeiten des Telenotarztes bei Großschadenslagen standen in Verbundprojekten wie VirtualDisaster [45] bereits im Fokus.

### Wissenschaftliche Kernaspekte und zukünftiger Forschungsbedarf

Die in Aachen durchgeführte DFG-geförderte randomisierte kontrollierte TEMS-Studie [36] diente dem Ziel, in einem hochwertigen durch Software randomisierten Studiendesign zu zeigen, dass der Telenotarzt hinsichtlich der klassischen telemedizinischen Notfallbilder nicht dem bodengebunden Notarzt unterlegen ist. Dies war zuvor in verschiedenen retrospektiven Auswertungen von TNA-Einsätzen bereits gezeigt worden. Einer der primären Endpunkte der TEMS-Studie war die Komplikationsrate. Die endgültigen Ergebnisse, welche die Nichtunterlegenheit des TNA-Systems darstellen, werden in Kürze publiziert. Aus der bisherigen telenotfallmedizinischen Forschung lassen sich die in Tab. 4 dargestellten Kernaussagen zusammenfassen.

Während es mittlerweile einige vergleichende Untersuchungen zwischen Notarzt und Telenotarzt gibt, die deren Gleichwertigkeit der Versorgungsqualität zeigen [7, 10, 11, 24], fehlen adäquate Studien zur Arbeitsweise von Rettungsteams ohne Arztbeteiligung. Studien mit Erfassung des Patient:innenzustands bei Übergabe an der Schnittstelle Notaufnahme gibt es kaum, und retrospektive Auswertungen der Dokumentation lassen Defizite in der Versorgung vermuten [23]. Es sind zwingend regelmäßige Schulungen und Überprüfungen des Rettungsdienstpersonals und auch der Notärzt:innen erforderlich, um eine optimale Zusammenarbeit aller verfügbaren Ressourcen, inklusive des Telenotarztes, zu erreichen und damit die Patient:innenversorgung zu verbessern bzw. deren Qualität zu sichern.

## Limitationen

Dieser Übersichtsartikel beschreibt die Erfahrungen des ersten und damit bisher am längsten im Regelrettungsdienst eingebundenen Telemedizinsystems der Stadt Aachen. Dies heißt jedoch nicht, dass die Aachener Vorgehensweise die einzig mögliche darstellt, vielmehr beschreibt sie einen erfolgreichen Weg der Anwendung von Telemedizin im Rettungsdienst.

## Fazit für die Praxis

Telenotfallmedizin

- stellt eine zeitgemäße und überregionale Ergänzung der notärztlichen Ressourcen dar und führt zu einem indikationsgerechteren Einsatz des Notarztes.
- fügt sich durch sorgfältige Abstimmung von Notarztindikationskatalog, möglicher Individualdelegation und eigenständigen Maßnahmen gemäß §4 Abs. 2 Nr. 2c NotSanG ins Gesamtsystem Rettungsdienst ein.
- führt zu einer Qualitätssteigerung im Rettungsdienst und kann prähospital in den unterschiedlichsten Einsatzbereichen unterstützen.
- bietet das Potenzial, (not-)ärztliche Expertise langfristig und flächendeckend standortunabhängig im Rettungsdienst zu verankern.
- wird aktuell bundesweit zunehmend in den Rettungsdienst integriert.
